# The Longitudinal Impact of Family, Religious, and School Support on the Mental Health of Filipino and Korean American Youth Across Adolescence

**DOI:** 10.1007/s40615-024-02200-z

**Published:** 2024-10-16

**Authors:** Michael Park, In Young Park, Yoonsun Choi, Julia R. Henly

**Affiliations:** 1 School of Social Work, Rutgers, The State University of New Jersey, New Brunswick, NJ 08901 USA; 2 School of Social Work, Boston College, Chestnut Hill, MA USA; 3 Crown Family School of Social Work, Policy and Practice, University of Chicago, Chicago, IL USA

**Keywords:** Social support from family, religious organization, and school, Depressive symptoms, Suicidal thoughts, Filipino American adolescents, Korean American adolescents

## Abstract

Asian Americans, currently the most rapidly expanding racial group in the USA, are experiencing persistent mental health inequities. These inequities are particularly pronounced during adolescence, highlighting the critical need for dedicated focus on this demographic. Social support is a key shield against mental distress among these adolescents, yet little research has examined the collective impact of multiple sources of support over time, across developmental stages, and for different ethnic subgroups. Drawing on the integrated conceptual framework, this longitudinal study is the first to our knowledge to examine how support from families, religious organizations, and schools together predicts mental health over time across adolescents’ developmental stages. We further examined how these relationships may be moderated by age groups, distinguishing between the early adolescence cohort and the middle adolescence cohort. Data were from the Midwest Longitudinal Study of Asian American Families (MLSAAF) project that survey-interviewed 378 Filipino American and 408 Korean American adolescents (*M*_age_ = 15 years). Findings reveal that family support consistently buffers against mental distress throughout adolescence for both groups. Religious support distinctly aids Filipino American early adolescents, while school support notably benefits early adolescents regardless of ethnicity. These findings emphasize the universal protective effects of social support, while also highlighting the nuanced ways that developmental stage and ethnicity may influence how different sources of support impact mental health. This underscores the need for developmentally and culturally sensitive mental health strategies for Asian American adolescents.

**Supplementary Information:**

The online version contains supplementary material available at 10.1007/s40615-024-02200-z.

## Introduction

Asian Americans, the fastest-growing racial group in the USA [1, 2], face enduring mental health inequities [3], especially during the critical period of adolescence [4, 5], underscoring an urgent need for focused attention on this group. Social support has been identified as an important protective factor against mental health struggles for racially minoritized adolescents, including Asian Americans [6–9]. However, research has not sufficiently examined the collective impact of multiple sources of social support in mitigating mental distress, despite evidence suggesting that not every source of support is equally efficacious [10–12]. Furthermore, no prior longitudinal study has investigated how the protective effects may vary over time across adolescent developmental stages. There is also a paucity of longitudinal research that dissects these trends within diverse Asian American subpopulations. This study is the first to our knowledge to examine the relationship between social support from families, religious organizations, and schools—which can uniquely affect Asian Americans in relation to their immigration status and racial minority status—and the experience of depressive symptoms and suicidal thoughts in Filipino American and Korean American adolescents. Additionally, it investigates how these protective effects change over a 4-year study period and how the effects further vary by adolescent developmental stages (i.e., early adolescence vs. middle adolescence at baseline).

### Mental Health Inequities Among Asian American Adolescents

Research has documented persistent inequities in mental health outcomes, notably depression and suicidality, not only among adolescents in general [13, 14] but also more acutely among racially minoritized adolescents. As these young individuals progress through their developmental years, mental health challenges escalate. For instance, studies indicate that mental health struggles begin to intensify around age 15 and typically peak during late adolescence or the transition into young adulthood [13, 14]. According to the 2021 National Survey on Drug Use and Health (NSDUH) [14], adolescents aged 12 to 17 reported the highest rates of major depressive episodes (20.1% or 5.0 million individuals), compared with the 18 to 25 age group (18.6%), and the 26 to 49 age group (9.3%). Similarly, the prevalence of suicidal thoughts among adolescents aged 12 to 17 (12.7% or 3.3 million) was nearly equivalent to that of the 18 to 25 age group (13.0%), but significantly higher than in the 26 to 49 age group (5.4%).

A range of socio-demographic factors significantly heightens the mental health risks for Asian American adolescents. Among these are the stresses associated with being a racially minoritized group, such as facing increased racial discrimination [15] and being perceived as perpetual outsiders [16]. Additionally, navigating life as children of immigrants introduces its own challenges. This often involves navigating the stresses of acculturation [17], as they attempt to reconcile their family's cultural heritage with that of the dominant society, leading to intergenerational cultural conflict with their immigrant parents [18]. Indeed, mental health struggles among Asian American adolescents are alarmingly high. For instance, 2017 NSDUH data indicate that at age 15, Asian American adolescents had the highest incidence of major depressive episodes (24.7%) compared with peers from other racial and ethnic groups, including multiracial individuals (23.6%), Hispanic Americans (19.8%), non-Hispanic White Americans (17.1%), and African Americans (12.4%) [3]. Although Asian American young adults aged 18 or older reported the lowest rates of suicidal thoughts (3.4%) in 2022 compared to other racial and ethnic groups, including multiracial individuals (9.3%), African Americans (5.5%), non-Hispanic White Americans (5.2%), and Hispanic Americans (4.6%) [19], suicide remains the leading cause of death for Asian Americans aged 15 to 24, which is a distinctive trend for this demographic [4]. Furthermore, between 2013 and 2019, the suicide rate among this group escalated by over 40% [5], underscoring a critical public health concern.

### Conceptual Framework

Building on previous cultural-ecological models [20], Mistry et al. [21] put forward an integrated conceptual framework for the development of children and youth, specifying dimensions that are particularly relevant to Asian American children and families based on their individual cultural backgrounds. This model posits that the development of Asian American adolescents is shaped by both general youth-relevant contexts (e.g., family, neighborhoods, and schools) and specific aspects related to Asian Americans, such as immigration status and social stratification. Specifically, as children of immigrants, Asian American children navigate between their parents’ culture of origin and US culture, while simultaneously facing challenges associated with being part of a minoritized group. Within this societal structure, various settings can either bolster or hinder mental health, depending on the accessibility of supports and alignment with their specific needs.

This research delves into three ecological settings critical for understanding Asian American adolescents, as suggested in the integrated model [21] and supported by empirical studies. It specifically investigates the impact of family, religious organizations, and schools—elements related to immigration and racially minoritized status—on the mental health of these adolescents. The role of the family is particularly significant, especially given that a majority of Asian Americans are immigrants; over 62% were born outside the USA [22]. This demographic detail underscores the distinct challenges and experiences faced by these Asian American immigrant families in reconciling their native country’s values and cultures with those of the USA. Being part of immigrant families, they experience acculturation as they adapt to mainstream society, while also maintaining the customs of their cultures of origin [23]. This dual process can often result in intergenerational cultural conflict, further complicating adolescents’ mental health challenges.

Religious institutions play a vital role in supporting immigrants, offering community and shared support [24]. Yet, these organizations can also exert stress by upholding hierarchical and patriarchal values, potentially conflicting with the more egalitarian ideals prevalent in wider American society [25]. Navigating these conflicting values can pose significant challenges for Asian adolescents, possibly manifesting in mental distress [26]. Although studies on religiosity across race and ethnicity report that Asian Americans are, on average, less religious than other racial and ethnic groups—for instance, only 26% of Asian Americans reported attending religious services at least once a week in 2014, compared to 34% of non-Hispanic White Americans, 39% of Hispanic Americans, and 47% of African Americans [27]—foreign-born Asian Americans are more religious than their US-born counterparts (32% vs. 21%) [28]. Furthermore, the role of faith-based support may be especially pertinent for Filipino American and Korean American communities, given their higher levels of religious affiliation compared to other Asian American communities (i.e., 92% and 77% compared to 68%, respectively).

In educational settings, Asian American students navigate complex racial stereotypes. They are often labeled as the “model minority,” a perception that characterizes them as hardworking and intelligent [29]. Concurrently, they grapple with being perceived as perpetual foreigners, an assumption that questions their belonging and acceptance as Americans [30]. These multifaceted stereotypes contribute to a challenging educational and social experience. For instance, Asian American students often encounter praise from educators for embodying the model minority image, yet they may also face social challenges, such as being viewed as socially awkward [31]. This stereotyping practice of teachers may foster resentment among other racially minoritized students, leading to peer harassment [32]. Indeed, according to the Centers for Disease Control and Prevention, when specifically asked about being treated unfairly at school due to race or ethnicity, Asian American students reported the highest incidence (64%) of racial discrimination among major racial groups [33]. This high incidence of discrimination could discourage them from seeking or receiving support within educational settings, particularly when they are in need of support.

As outlined in cultural-ecological perspectives [20], the support and opportunities that families, religious organizations, and schools offer to minoritized adolescents are shaped by both macrotime cultural shifts and developmental needs at the given time. Macrotime [34] refers to societal changes occurring within and across generations. For instance, changes in the sociopolitical landscape regarding Asian Americans or alterations in family dynamics and roles can significantly modify the nature and accessibility of support adolescents receive from the surrounding ecological environment. More importantly, the support and opportunities offered to minoritized adolescents are also likely to vary across adolescent developmental stages. According to the life course perspective [35–37], individuals’ life stages are shaped by the interplay of age-graded transitions, social institutions, culture, and historical contexts, with adolescent development being influenced by external support systems such as family, schools, and religious organizations. For instance, during early adolescence, familial support is typically more crucial than later developmental stages, as this period is marked by dependence on immediate caregivers and close family relationships [36]. Adolescents at this stage may rely on familial guidance to navigate developmental challenges, including mental health concerns [38]. Yet, as children transition to middle and late adolescence, the life course perspective highlights a shift in social priorities, with adolescents increasingly relying on and gaining greater benefits from support provided by other ecological systems such as religious organizations and schools [39]. This shift reflects the broadening of social networks and the adolescent’s growing independence from the family. While these existing theoretical frameworks highlight the significance of macrotime changes in environmental affordances and the varying effects of ecological systems across developmental stages, few studies have investigated how the influence of social support from key ecological systems—like families, religious organizations, and schools—evolves over time across developmental stages. In this research, we delve into these relationships using a three-wave longitudinal study spanning from 2014 to 2018, with a specific emphasis on the developmental stages of early and middle adolescence.

Our study specifically focuses on Filipino American and Korean American adolescents. The integrated conceptual framework [21] posits that surrounding ecological systems are steeped in culture, leading to varied experiences based on individual cultural backgrounds. Although Filipino Americans and Korean Americans share some social characteristics, including socioeconomic status and racially minoritized status, they are significantly different in immigration history, acculturation experiences, family process, and health outcomes [16, 17]. Therefore, rather than grouping Filipino American and Korean American under a broad Asian American category, we investigate the unique experiences of these ethnic subgroups.

### Social Support and Mental Health in Adolescence

Social support generally refers to a broad range of resources transferred across social relationships [40]. Significant evidence points to families [7], religious organizations [8, 9], and schools [6] as critical sources of support that help protect mental health. However, studies have often focused on family and school support, with less attention given to the effects of religious support. Moreover, the relationship between these support systems and mental health outcomes has been inconsistent. Some studies have demonstrated that family and school support can together mitigate depression [41], anxiety [6], and suicidal thoughts [42], yet others found that stronger school relationships could elevate risk for suicide attempts [7]. Systematic reviews also reflect this complexity. For example, in their systematic review of 121,432 nonimmigrant children and adolescents, Chu et al.[43] noted that teacher support was a stronger correlate of well-being than family support. In contrast, Rueger et al. [44] found that, in a study encompassing 273,430 children and adolescents, family support was the most predictive of depressive symptoms, followed by teacher support.

The inconsistent findings may partly stem from not considering support sources collectively. For instance, while religious support is recognized as a significant protective factor for Asian American adolescents [8, 9], no prior study has examined it alongside other sources of support. The meta-analysis by Rueger et al. [44], for example, categorized religious support under “miscellaneous” with other sources, which might mask its specific effects. Given its particular importance to Asian Americans, this study includes religious support, in addition to other critical support sources, to get a clearer picture of its effect on adolescent mental health.

### The Effects of Social Support Over Time by Adolescent Age Groups

The differences in outcomes from various sources of support may be also due to age differences, as adolescents’ needs and interactions with their environment change between early and middle adolescence. To date, research, not only on Asian American adolescents but also on adolescents in general, has not specifically looked at how the effects of multiple sources of social support shift over time and across different adolescent age groups (e.g., the interactions between social support sources, specific time points, and age groups). Among the handful that looked into how multiple sources of support affect specific age groups, Arora et al. [45] found that for Asian American early adolescents, teacher support, not parental support, was a consistent predictor of reduced depressive symptoms. Conversely, in their study of Black early adolescents, McMahon et al. [10] found that support from parents, but not from teachers and classmates, was significantly associated with lower anxiety and depression. In the case of middle adolescents, Yeh et al. [6] observed with Chinese immigrant high school students that both family and school support were beneficial in reducing depression and anxiety. Colarossi and Eccles [11] found in their study of mostly White (92%) middle adolescents that support from peers and teachers was predictive of lower depression and higher self-esteem, and support from mothers was associated only with depression. Another study found that neither parent, teacher, nor peer support had protective effects on depressive symptoms of Korean immigrant middle adolescents [46].

On the other hand, Gariepy et al. [39] conducted a comprehensive review of sources of support influencing depression, involving over half a million children and adolescents without a specific focus on ethnicity or age groups, and found that parental support was most critical for children and adolescents, whereas spousal, family, and friend support was more influential for adults. These divergent findings regarding sources of social support and mental health outcomes among adolescents could be due to a research gap in tracking how the influence of social support changes over time within different adolescent age brackets [44]. The current study bridges these gaps by concentrating on the longitudinal dynamics of social support across adolescent age groups.

## The Current Study

Drawing from the integrated conceptual framework [21], this longitudinal study of Filipino American and Korean American adolescents explored how social support from families, religious organizations, and schools jointly predict depressive symptoms and suicidal thoughts (main effect), how these protective effects change over three waves from 2014 to 2018 (two-way interaction effects between support and wave), and, further, how these relationships may be moderated by age groups (three-way interaction effects between support, wave, and age groups). Age groups were defined as an early adolescence cohort (middle schoolers at baseline) and a middle adolescence cohort (high schoolers at baseline). First, we expected that family, religious, and school support would all predict fewer mental health struggles, independent of ethnic background. We did not formulate separate hypotheses for depressive symptoms versus suicidal thoughts, as we anticipated consistent findings across these dimensions based on existing literature. Secondly, while protective effects of family support were expected to wane over time for both ethnic groups, the benefits of religious and school support were anticipated to grow particularly among Korean American adolescents. This group, often coming from less acculturated backgrounds than other Asian American immigrants in the USA, may turn more toward external sources of social support (such as religious and school support) as they advance through adolescence. Given the scarcity of existing research, hypotheses regarding three-way interaction effects between support, wave, and adolescent developmental stages are largely exploratory. Nevertheless, a more significant decline in family support's protective effects was anticipated for the older adolescents (the middle adolescence cohort) compared with the younger ones (the early adolescence cohort), irrespective of ethnicity. Similarly, the protective effects of religious and school support were expected to have a more substantial increase among older adolescents, especially within the Korean American group.

## Methods

### Participants and Procedure

Data for this study were derived from the Midwest Longitudinal Study of Asian American Families (MLSAAF) project, an ongoing survey of Filipino American and Korean American children and their parents living in a Midwest metropolitan area. The MLSAAF project specifically selected Filipino and Korean American adolescents to ensure socioeconomic comparability while capturing variability in key socio-cultural factors [15–17, 40]. These subgroups were chosen to explore both shared and distinct experiences relevant to mental health outcomes. Further rationale for this selection can be found in prior studies [15–17]. The present study used the first three waves (i.e., 2014 to 2018) of the child data. The first wave (W1) was collected in 2014 from 378 Filipino American and 408 Korean American adolescents (*N* = 786). The data at the second wave (W2) were collected in 2016 with a retention rate of approximately 78% relative to W1 (*N* = 653; 302 Filipino American and 351 Korean American adolescents). The third wave (W3) surveys were collected in 2018, with 309 Filipino American and 341 Korean American adolescents (*N* = 650, 81% retention rate relative to W1). The questionnaires were available in both paper and online formats and were provided in English, Korean, and Tagalog. More details about data collection and procedures are available elsewhere [17]. The average age of participants at W1 was 15.3 years (*SD* = 1.88) for Filipino American and 14.8 years (*SD* = 1.91) for Korean American adolescents, with a larger proportion of high school students (78.7% of Filipino American and 75.3% of Korean American adolescents) than middle school students. Gender distribution was about equal, with 56% of the Filipino American and 47% of the Korean American adolescents identifying as female. A total of 74.4% of the Filipino American and 59.6% of the Korean American adolescents were US-born (see Table S1 for further details).

### Measures

#### Adolescent Outcomes

*Depressive symptoms* were based on 14 items from the Children’s Depression Inventory [47] and the Seattle Personality Questionnaire for Children [48]. Adolescents were asked to rate depressive symptoms experienced in the past two weeks on an ordinal scale (1 = *almost never* to 5 = *almost always*). Examples of the items include “I didn’t enjoy anything at all” and “I was very restless” (α ranging from 0.93 to 0.94 for Filipino Americans and Korean Americans across three waves).

*Suicidal thoughts* were measured by a single item that asked adolescents whether they had ever seriously thought about committing suicide during the past 12 months, rated on a dichotomous scale (0 = *No*, 1 = *Yes*).

#### Social Support

Three scales were used to measure support from surrounding ecological systems. *Family support* was measured by using nine items developed by Wu and Chao [49] that assessed parental instrumental support, e.g., “My parents invest all what they have for my education,” and “My parents know all my possible needs before I am aware of them” (α from 0.85 to 0.90 for Filipino American and from 0.84 to 0.90 for Korean American across the three waves). *Religious support* was measured with two items from the MLSAAF, assessing perceived support from religious places including church or a place of worship: “There is at least one adult at church (or a place of worship) that I can talk to if I have a problem,” and “Friends at church (or a place of worship) who are not in my school help me do my best” (*r* ranges from 0. 39 to 0.43 for Filipino American and from 0.27 to 0.40 for Korean American across the three waves). *School support* included two items from the MLSAAF that are related to perceived school support: “There is at least one teacher or other adult at school that I can talk to if I have a problem” and “Other students in my class help me do my best” (*r* ranges from 0.39 to 0.43 for Filipino American and from 0.27 to 0.40 for Korean American across the three waves). Response options were on an ordinal Likert scale ranging from 1 to 5.

#### Covariates

Several adolescent demographic variables were included as covariates. Demographic characteristics include biological sex (male vs. female), nativity (foreign-born vs. US-born), adolescent developmental stages (early adolescence cohort [middle schoolers at baseline] vs. middle adolescence cohort [high schoolers at baseline]), adolescent-reported household socioeconomic status (1 = *lower class* to 5 = *upper class*), and general health condition (1 = *very poor* to 5 = *very good*).

#### Analysis Steps

Descriptive analyses examined participants’ general characteristics and differences across adolescent ethnicity and developmental stages. Bivariate correlations among the main study variables were examined within each ethnic group.

Using Stata v. 17.1, this study used linear mixed-effect regression analyses to estimate longitudinal effects of social support on mental health outcomes over developmental stages. Mixed-effect regression models can be applied to both normally distributed continuous outcomes and categorical outcomes [50], are robust to missing data, and can easily handle both time-varying (e.g., social support) and time-invariant (e.g., nativity) covariates. This study used mixed effect linear regression for continuous variables (e.g., depressive symptoms) and mixed effect logistic regression models for binary outcome variables (e.g., suicidal thoughts).

The models used three hierarchical steps for testing direct effects, two-way interactions, and three-way interactions. First, the direct effect model (model 1) included main predictor variables (i.e., family support, religious support, and school support) and covariates. In addition, we disentangled the direct effects of each social support source into between- and within-subjects effects to provide a clearer picture of between- and within-person processes [50]. The estimates of the between-subjects effect use the average of the samples across the waves, whereas estimates of the within-subjects effect compare each individual participant in the sample at a given time point to their own average across the waves [15]. We conducted the likelihood ratio (LR) test to compare goodness of fit between models without and with the decomposition of the social support. If the LR test indicated no significant difference between the two models, we used the model without the decomposition. On the other hand, if the LR test comparing two models resulted in any statistically significant difference, we used the models that include the between- and the within-subjects social support. In the two-way interaction model (model 2), we examined whether the effects of social support change over time or developmental stages; thus, two-way interaction terms (family support × wave or developmental stages, religious support × wave or developmental stages, school support × wave or developmental stages) were added to the direct effect model. Lastly, in the three-way interaction model (model 3), we added three-way interaction terms (e.g., social support × developmental stages × wave) to the two-way interaction models. Specifically, the three-way interaction terms—(1) family support × wave × developmental stages, (2) religious support × wave × developmental stages, and (3) school support × wave × developmental stages—were added and tested separately. The interaction was then probed further by plotting slopes following the procedures outlined by Dawson and Richter [51] and comparing these slopes for the early versus middle adolescence cohorts when social support was high (vs. low). All these analyses were conducted within each ethnic group.

The percentage of missing data across all study variables was less than 5% (ranging from 0.0 to 2.7% for Filipino Americans and from 0.0 to 2.9% for Korean Americans). We used the maximum likelihood estimation method [50] to handle missing data. All variables had correlation coefficients in the range of 0.00 to 0.41, less than the cutoff value of ± 0.80 for multicollinearity, and the variance inflation factor values (< 10) showed no evidence of multicollinearity for all study variables. We excluded samples with no religious affiliation information given that the measure pertaining to religious support was only asked of those who reported their religious affiliation. Specifically, 9.5% of Filipino American and 14.3% of Korean American did not have religious affiliation at W1 (see Table S1).

## Results

### Descriptive Statistics

Table 1 presents the correlations between the study variables at each wave. Table S1 additionally provides the study variable means and sample sizes broken down by ethnicity and developmental stages. We used an independent-samples *t*-test and chi-square test to examine statistical differences by ethnicity and developmental stages. We found significant differences in social support sources by ethnicity across all three waves (Table S1). Specifically, Filipino American adolescents reported significantly higher family and school support compared with their Korean American counterparts, whereas Korean American adolescents reported significantly higher religious support. There were no differences in depressive symptoms or suicidal thoughts based on ethnicity. Regarding developmental stages, the early adolescence cohort reported lower rates of depressive symptoms and suicidal thoughts than the middle adolescence cohort.

**Table 1:** Mixed-effects regression results for depressive symptoms among Filipino American adolescents

	Depressive symptoms
	*b* (*SE*)
Model 1	
Wave	0.19 (0.04)^***^
Baseline developmental stages	0.32 (0.08)^***^
Wave × baseline developmental stages	− 0.15 (0.05)^**^
Nativity (0 = foreign − born; 1 = US − born)	− 0.12 (0.07)
Biological sex (0 = male; 1 = female)	0.27 (0.07)^***^
Family socioeconomic status	− 0.05 (0.04)
General health	− 0.26 (0.03)^***^
Family support (between)	− 0.26 (0.06)^***^
Family support (within)	− 0.11 (0.05)^*^
Religious support	− 0.07 (0.03)^**^
School support	− 0.08 (0.03)^*^
Model 2 (two − way)	
Family support (between) × developmental stages	− 0.13 (0.12)
Family support (within) × developmental stages	0.06 (0.10)
Religious support × developmental stages	0.05 (0.05)
School support × developmental stages	0.08 (0.07)
Model 3 (three − way)	
Family support × developmental stages × wave	0.04 (0.08)
Religious support × developmental stages × wave	0.11 (0.05)^*^
School support × developmental stages × wave	0.07 (0.07)
Observations	767
Number of groups	334

Support (between) = the between − subjects effect of social support; support (within) = the within − subjects effect of social support. The findings from model 1, model 2, and model 3 are combined in such a way that the top of the column shows coefficient from model 1 and the bottom of the column shows interaction coefficients from model 2 and model 3

^***^p < .001; **p < .01; *p < .05; ^+^*p* < .1

### Direct Effects Models

While accounting for demographic control variables (including biological sex, nativity, adolescent developmental stages, adolescent-reported household socioeconomic status, and general health condition), family support, religious support, and school support were regressed together on depressive symptoms (see Table 1 and Table 2) and on suicidal thoughts (see Table 3 and Table 4). For the Filipino American adolescent group, all social support sources, including family, religious, and school support predicted lower depressive symptoms. Specifically, both between-subjects (*b* =  − 0.26, *p* < 0.001) and within-subjects (*b* =  − 0.11, *p* < 0.05) effects of family support on depressive symptoms were statistically significant. This indicates that family support was higher than the average for the Filipino American adolescents across the three waves (i.e., the between-subjects effect) and that an increase in family support within individual adolescents over time (i.e., within-subjects effect) reduced depressive symptoms. For the Korean American adolescent group, family and school support were significant predictors of depressive symptoms (Table 2). Specifically, we found significant between-subjects (*b* =  − 0.28, *p* < 0.001) and within-subjects (*b* =  − 0.12, *p* < 0.01) relations between school support and the level of depressive symptoms. Regarding suicidal thoughts, for the Filipino American adolescents, a higher rate of the between-subjects effect of family support was associated with fewer suicidal thoughts (*OR* = 0.20, *p* < 0.001; Table 3). For Korean American adolescents, school support predicted fewer suicidal thoughts (*OR* = 0.60, *p* < 0.05; Table 4).

**Table 2:** Mixed-effects regression results for depressive symptoms among Korean American adolescents

	Depressive symptoms
	*b* (*SE*)
Model 1	
Wave	0.18 (0.04)^***^
Baseline developmental stages	0.32 (0.07)^***^
Wave × baseline developmental stages	− 0.11 (0.05)^*^
Nativity (0 = foreign − born; 1 = US − born)	0.14 (0.06)^*^
Biological sex (0 = male; 1 = female)	0.18 (0.06)^**^
Family socioeconomic status	− 0.03 (0.04)
General health	− 0.16 (0.03)^***^
Family support (between)	− 0.08 (0.06)
Family support (within)	− 0.13 (0.05)^*^
Religious support	− 0.02 (0.03)
School support (between)	− 0.28 (0.06)^***^
School support (within)	− 0.12 (0.04)^**^
Model 2 (two-way)	
Family support (between) × developmental stages	− 0.06 (0.11)
Family support (within) × developmental stages	− 0.01 (0.11)
Religious support × developmental stages	0.01 (0.05)
School support (between) × developmental stages	− 0.08 (0.10)
School support (within) × developmental stages	0.16 (0.07)^*^
Model 3 (three-way)	
Family support × developmental stages × wave	0.00 (0.08)
Religious support × developmental stages × wave	− 0.03 (0.06)
School support × developmental stages × wave	0.08 (0.07)
Observations	798
Number of groups	349

Support (between) = the between − subjects effect of social support; support (within) = the within − subjects effect of social support. The findings from model 1, model 2, and model 3 are combined in such a way that the top of the column shows coefficient from model 1 and the bottom of the column shows interaction coefficients from model 2 and model 3

^***^p < .001; **p < .01; *p < .05; ^+^*p* < .1

**Table 3:** Mixed-effects regression results for suicidal thoughts among Filipino American adolescents

	Suicidal thoughts
	*OR*
Model 1	
Wave	1.12
Baseline developmental stages	0.53
Wave × baseline developmental stages	1.23
Nativity (0 = foreign − born; 1 = US − born)	0.89
Biological sex (0 = male; 1 = female)	2.66^*^
Family socioeconomic status	0.67
General health	0.55^**^
Family support (between)	0.20^***^
Family support (within)	1.35
Religious support	0.83
School support	0.69^+^
Model 2 (two-way)	
Family support × developmental stages	0.93
Religious support × developmental stages	1.67^+^
School support (between) × developmental stages	1.95
School support (within) × developmental stages	0.23^**^
Model 3 (three-way)	
Family support × developmental stages × wave	0.98
Religious support × developmental stages × wave	1.05^+^
School support × developmental stages × wave	1.02
Observations	762
Number of groups	333

Support (between) = the between − subjects effect of social support; support (within) = the within − subjects effect of social support. The findings from model 1, model 2, and model 3 are combined in such a way that the top of the column shows coefficient from model 1 and the bottom of the column shows interaction coefficients from model 2 and model 3

*OR* odds ratio

^***^p < .001; **p < .01; *p < .05; ^+^*p* < .1

**Table 4:** Mixed-effects regression results for suicidal thoughts among Korean American adolescents

	Suicidal thoughts
	*OR*
Model 1	
Wave	2.01^*^
Baseline developmental stages	4.28^*^
Wave × baseline developmental stages	0.62
Nativity (0 = foreign − born; 1 = US − born)	1.24
Biological sex (0 = male; 1 = female)	1.99^+^
Family socioeconomic status	0.88
General health	0.83
Family support	0.79
Religious support	0.97
School support	0.60^*^
Model 2 (two-way)	
Family support × developmental stages	1.21
Religious support × developmental stages	0.90
School support × developmental stages	0.90
Model 3 (three-way)	
Family support × developmental stages × wave	0.99
Religious support × developmental stages × wave	0.99
School support × developmental stages × wave	0.98
Observations	794
Number of groups	350

Support (between) = the between − subjects effect of social support; support (within) = the within − subjects effect of social support. The findings from model 1, model 2, and model 3 are combined in such a way that the top of the column shows coefficient from model 1 and the bottom of the column shows interaction coefficients from model 2 and model 3

*OR* odds ratio

^***^p < .001; **p < .01; *p < .05; ^+^*p* < .1

### Two-Way Interaction Models

The results for the two-way interactions between social support and wave indicated that, for both ethnic groups, the protective effects of social support on depressive symptoms did not vary across waves. As for interaction effects between social support and adolescent developmental stages (Table 1), significant interaction effects were found only among Korean American adolescents. Specifically, developmental stages significantly moderated the association between school support (the within-subjects effect) and depressive symptoms among Korean American adolescents (*b* = 0.16, *p* < 0.05; Table 2; Figure S1). Specifically, a follow-up slope test showed that the within-subjects effect of school support was associated with fewer depressive symptoms for Korean American adolescents, and this relationship was stronger for those in the early adolescence cohort than for those in the middle adolescence cohort. For the models predicting suicidal thoughts (Table 3), school support (the within-subjects effect) predicted fewer suicidal thoughts for the Filipino American early adolescence cohort (vs. middle adolescence cohort) (*OR* = 0.23, *p* < 0.01; Figure S2). None of the interaction effects was significant among Korean American adolescents.

### Three-Way Interaction Models

We found significant interaction effects between religious support, wave, and developmental stages on depressive symptoms only among Filipino American adolescents (*b* = 0.11, *p* < 0.05; Figure S3). A follow-up slope test showed that depressive symptoms significantly increased over time among the early adolescence cohort, regardless of their level of religious support. However, the protective effect of religious support on depressive symptoms was stronger for those who reported higher religious support (1 *SD* above the mean) than for those who reported religious support at the mean or lower. Among the middle adolescence cohort, we found that depressive symptoms did not significantly increase over time and that the effects of religious support did not vary by its level (1 *SD* below the mean vs. the mean vs. 1 *SD* above the mean). Regarding suicidal thoughts, we found no significant three-way interaction effects regardless of ethnicity.

### Alternate Models

We conducted a sensitivity analysis to investigate whether the tested main and interaction effects of social support from multiple sources provide a sufficient explanation of how each source is independently related to depressive symptoms and to suicidal thoughts. This involved analyzing the three-way interaction effects among all support types (family support × religious support × school support) in relation to each mental health outcome. The absence of significant three-way interaction terms for both adolescent ethnic groups suggests that each support source contributes independently to the explanation of these mental health outcomes.

## Discussion

Social support is a critical factor in shaping the mental health of Asian American adolescents. Nevertheless, there is a research gap regarding the combined and temporal effects of diverse social support sources on mental health outcomes throughout adolescence. Drawing on the integrated conceptual framework [21], our study pioneers in examining the longitudinal impact of key ecological support systems—namely families, religious organizations, and schools—on Filipino American and Korean American adolescents. This research departs from earlier work that concentrated on predicting the effect of early social support on subsequent mental distress (e.g., utilizing simple path models) and studies examining how average effects of social support are linked with mental health outcomes across sample averages (between-subjects effects). Instead, our investigation encompasses both between- and within-subjects effects to provide a more comprehensive longitudinal analysis of social support’s influence on mental health outcomes. Furthermore, there has been a call from developmental scientists to focus on subgroup differences and the confluence of multiple social identities [20, 21]. In response, our study not only delves into the variations across developmental stages—comparing cohorts of early and middle adolescents—but also examines these dynamics across different ethnicities.

Overall, the findings indicate that even after accounting for key demographic characteristics such as biological sex, nativity, and socioeconomic status, social support continues to play a broadly protective role in the mental health of Asian American adolescents across developmental stages and ethnicity, with some variations depending on specific sources of support and contextual factors. Specifically, family support consistently served as a safeguard against mental distress over time and across development stages in both ethnic groups studied. While the role of religious and school support may vary, these sources of support remain invaluable across different contexts. That is, religious support appeared particularly beneficial for the Filipino American early adolescence cohort, while school support was a significant factor positively impacting the mental well-being of the early adolescents in both ethnic groups, underlining the essential role that schools play in supporting the mental health of younger adolescents. These findings underscore the universal importance of social support as a protective factor across ages and groups, while also acknowledging some nuanced variations in how different sources of support may function. The study emphasizes the need to consider both shared developmental experiences and specific contextual factors when addressing the mental health needs of Asian American adolescents, advocating for an approach that is sensitive to both developmental stage and ethnicity.

As expected, for both ethnic groups, family support emerged as a significant predictor of depressive symptoms, even after taking other support sources into consideration. Notably, increased family support also predicted a reduction in suicidal thoughts among Filipino American adolescents. Contrary to our expectations, the protective effects of family support remained constant over time, holding significance across developmental stages irrespective of ethnicity. This observation challenges some existing literature that posits the diminishing influence of family support as adolescents mature [52]. A possible explanation could be that many current studies do not concentrate on the experiences of Asian American adolescents and instead focus on other racial groups, such as Black [10] or White [11] adolescents, or they consider Asian Americans as a monolithic group without recognizing differences among subgroups [45]. However, for Asian Americans, of whom over 62% are foreign-born [22], the family may remain a vital ecological setting that continuously provides resources or induces stress, contingent on the quality of familial relationships [15, 53]. Additionally, few existing studies use longitudinal datasets. Aligning closely with the current study findings, though based on a non-US cohort, Pössel et al. [12] discovered in their 5-year longitudinal study of 1452 Australian adolescents (aged 11–16 at baseline) that consistent family support across grades 8 to 12—and teacher support between grades 8 to 10—but not peer support, was linked to decreased depressive symptoms a year later.

The findings partially corroborate our hypotheses, demonstrating that religious support served as a protective factor against depressive symptoms but only among Filipino American adolescents and specifically among the early adolescence cohort. Depressive symptoms were observed to notably escalate among the early adolescence cohort but not among the middle adolescence cohort within the Filipino American group. However, this escalation in depressive symptoms was mitigated by the influence of religious support, underscoring the significant role religious organizations play within this community. Contrary to our expectations, religious support did not significantly predict suicidal thoughts for Filipino American adolescents, despite the literature suggesting its importance in preventing suicidal behaviors [54]. This result does not necessarily imply that religious support is unimportant; rather, its influence may be relatively weaker when compared to the more central roles of family and school support [41] in addressing severe mental health struggles, such as suicidal thoughts. Similarly, for Korean American adolescents, other support systems like family and school appear to be more instrumental in protecting against depressive symptoms and suicidal thoughts. Despite initial correlations between religious support and mental health outcomes, the significance diminished in the regression model after accounting for these additional support sources. Particularly in close-knit Korean American church communities, religious institutions may serve dual roles, acting not only as sources of support but also as potential sources of stress [26], thereby complicating their function in mental health support. Thus, religious support plays a nuanced role in mental health trajectories, varying in influence across different cultural and community contexts.

In line with our hypothesis, school support was directly linked to reduced mental health struggles across ethnicities, even after accounting for other sources of support. Unexpectedly, the protective effect of school support was pronounced in early adolescence. For instance, school support’s protective role against depressive symptoms for Korean American early adolescents and against suicidal thoughts for Filipino American early adolescents was absent among the middle adolescence cohort. As children transition into adolescence or middle school, they may cultivate a diverse array of new social relationships, distinct from those formed during their middle childhood. This period also brings a significant shift in socio-cognitive development, enabling racially minoritized adolescents to gain a more nuanced understanding of intricate social processes, including those associated with race and racism [55]. Indeed, empirical research indicates that perceptions of discrimination tend to escalate throughout adolescence [15]. Hence, during early adolescence, when individuals begin navigating these transitions, there might be an increased need for supportive school environments. As they move into middle or late adolescence, having potentially honed social skills and developed strategies to cope with discrimination, Asian American adolescents may find the impact of school support to be less significant than in earlier adolescence, at least in the case of mental health outcomes. Alternatively, it is possible that the school support measure used in this study may not be robust enough to fully capture the increasing and evolving support needs that adolescents experience as they grow older and face more complex mental health challenges. Future research should explore this further by utilizing more comprehensive and nuanced measures of school support to better assess its role across different developmental stages.

Notably, we found that school support was the only significant protective factor against suicidal thoughts among Korean American adolescents, regardless of their developmental stages. It is possible that family support may be more effective in addressing day-to-day emotional challenges closely tied to depressive symptoms, while schools may provide instrumental and informational support that helps at-risk adolescents cope with challenges (e.g., preparing for college or resolving conflicts with peers at the school setting) that cannot be fully resolved within the family system [56–58]. This finding is particularly significant for Korean American families, as parents in these families tend to be less acculturated to US mainstream culture [59] and may also face more difficulties navigating the US education system compared to other Asian American parents [57, 58], which increases the need for their children to seek support and guidance from teachers and peers.

### Limitations

Several limitations of this study must be noted. First, although our study aims to explain the effects of social support among adolescents by considering its source, our understanding may further benefit from considering other types of social support, such as informational and appraisal support [44]. However, this study comprehensively examined the intersection of different sources of support, macrotime cultural changes, developmental stages, and ethnicity. Introducing additional variables might not be practical due to the limited sample sizes of each intersectional group, and it could complicate the interpretation of the findings. Future research exploring the interactive effects of numerous factors would necessitate larger samples in an extended longitudinal study. Second, our measures of school and religious support, available from the MLSAAF data, rely on two-item scales. Furthermore, family support, as measured in the current study, assesses parental investment in the child’s education and the parents’ ability to anticipate their child’s needs. These are important aspects of parental support but differ from more commonly used measures in the literature, such as parental warmth and engagement [41]. Similarly, one of our school support items assesses the presence of a trusted adult at school. While this item is a valuable indicator of support within school environment, it may not fully capture the broader school context or school culture. Therefore, they may not align with the constructs typically measured in other research, and the findings may reflect more specific aspects of support rather than the general support structures often examined. Despite this limitation in these measures of support, this study is pioneering in its exploration of the longitudinal effects of various sources of social support on Filipino American and Korean American adolescents across different developmental stages. Future research should aim to validate the findings by employing more robust and comprehensive social support measures. Lastly, the generalizability of the results is limited as the participants were Filipino Americans and Korean Americans living in the Chicago metropolitan area. Given the diversity of Asian American subgroups in their immigration backgrounds and unique social support systems, additional research involving more ethnically diverse adolescent groups in other locations can enhance the generalizability of the obtained results.

Despite these limitations, the current study provides significant theoretical and practical implications for the development of Asian American adolescents. Various developmental theorists have suggested that conventional developmental theories, primarily based on the experiences of White adolescents, may not be wholly applicable to the unique experiences of Asian American adolescents [20, 21]. Much of the existing research either focuses predominantly on other racial groups or relies on cross-sectional data or limited longitudinal data [10, 11]. Expanding the depth and breadth of our understanding, the current study underscores the necessity of a nuanced approach in applying developmental theories, emphasizing the unique trajectories and challenges faced by Asian American adolescents. Furthermore, limited research has investigated the variation in effects of social support sources as racially minoritized children transition into higher education and/or emerging adulthood. Asian American children of immigrants, when entering this phase, may encounter unique challenges and developmental tasks that differ from those encountered in earlier developmental stages or those faced by other racial groups or even by their immigrant parents. There is a significant need for future research to delve deeper into this crucial topic.

This study also highlights the critical nature of early adolescence as an essential juncture for intervention, emphasizing the positive impact of support from religious organizations and schools during this developmental stage. The current study suggests that for Filipino American adolescents, substantial religious support is effective in reducing the rise in depressive symptoms during early adolescence. That is, for Filipino American adolescents, a significant increase in depressive symptoms was documented during early adolescence. This trend, however, was lessened by strong religious support. Notably, support from schools benefits early adolescents universally, regardless of ethnicity, underscoring the importance of nurturing positive relationships within educational settings to help prevent mental health struggles. Broad prevention efforts could include school- or classroom-wide assessments to gauge the level of support adolescents perceive from peers and educators, thus identifying those who may lack adequate support. Additionally, providing professional development for teachers to deepen their understanding of the unique challenges and mental health concerns faced by Asian American adolescents can be particularly valuable. Studies report that the model minority stereotype in schools often prevent educators from recognizing the needs and support gaps of underserved Asian American students, especially those lacking sufficient parental support [60, 61]. Therefore, while fostering an inclusive learning environment is essential, additional efforts are needed to dispel the model minority myth through targeted interventions for educators and school counselors working closely with Asian American adolescents. Such interventions could include training programs aimed at increasing awareness of racial dynamics and developing a deeper understanding of cultural differences [62]. Furthermore, strategically bolstering adolescents’ sense of support is key. This requires demonstrating a sincere commitment to student well-being [63], which is vital in devising effective intervention strategies. Considering the particular significance of religious and school support for mental health, the implementation of proactive and comprehensive support networks is essential. Establishing initial communication through existing religious organization and school channels can provide insight into the specific challenges and mental health needs of adolescents. Proactively providing such support structures can play a pivotal role in building resilience and preventing the development of mental health issues among early adolescents.

## Conclusion

Asian American adolescents, straddling their immigrant heritage and their minority status, face distinct challenges that can increase mental distress as they age. Existing research has not adequately explored the most beneficial sources of support during this pivotal period. Our study reveals that family support is vital for both Filipino American and Korean American adolescents, regardless of their developmental stage. While our findings indicate some variation in the influence of religious and school support, these sources of support remain invaluable across different contexts. Comparing the impact of support sources across adolescents’ developmental stages, our results underscore the importance of religious support for younger Filipino American adolescents, as well as the role of schools for younger adolescents across both ethnic groups. Overall, the study underscores the essential role of social support in promoting the mental health of minoritized adolescents as they transition from early to middle adolescence. We intend for our study to prompt further exploration into the diverse support systems that can bolster the mental health of minoritized adolescents as they advance toward adulthood.

## Supplementary Information

Below is the link to the electronic supplementary material.

Supplementary file1 (DOCX 361 KB)

## Data Availability

The datasets analyzed in the current study are not publicly available but can be available from the third author if certain conditions are met.
