# Supplementary material for: The Longitudinal Impact of Family, Religious, and School Support on the Mental Health of Filipino and Korean American Youth Across Adolescence
Source: J Racial Ethn Health Disparities. 2024 Oct 16;12(6):4053–66. doi: 10.1007/s40615-024-02200-z (PMC12644191; doi:10.1007/s40615-024-02200-z)
Supplement: Supplementary file 1 — Supplementary file1 (DOCX 361 KB) [file 40615_2024_2200_MOESM1_ESM.docx]

**Table S1**

*Descriptives of the samples*

|  | **FA** | | | | | **KA** | | | | | **Diff.** |
| --- | --- | --- | --- | --- | --- | --- | --- | --- | --- | --- | --- |
| Variables | FA Foreign-born (FF) | FA U.S.-born (FU) | FA Early adolescent | FA Middle adolescent | All FAs | KA Foreign-born (KF) | KA U.S.-born (KU) | KA Early adolescent | KA Middle adolescent | All KAs | All FAs vs. All KAs |
|  |  |  |  |  |  |  |  |  |  |  |  |
| **Demographic Characteristics** |  |  |  |  |  |  |  |  |  |  |  |
| Sample Sizes (W1) [*n* (%)] | 109(28.84%) | 269(71.16%) | 165(43.65%) | 213(56.35%) | 378(100%) | 171(41.91%) | 237(58.09%) | 215(52.70%) | 193(47.30%) | 408(100%) | N/A |
| Sample Sizes (W2) [*n* (%)] | 77(27.30%) | 202(71.63%) | 112(40.43%) | 165(59.57%) | 282(100%) | 127(38.72%) | 198(60.37%) | 171(52.62%) | 154(47.38%) | 328(100%) | N/A |
| Sample Sizes (W3) [*n* (%)] | 81(26.30%) | 223(72.40%) | 128(42.11%) | 176(57.89%) | 308(100%) | 136(40.00%) | 201(59.12%) | 170(50.45%) | 167(49.55%) | 340(100%) | N/A |
| Age (W1) | 15.45(1.81) | 15.20(1.90) | 15.05 (2.00) | 15.44 (1.76) | 15.27(1.88) | 15.23(1.94) | 14.42(1.81) | 14.74 (1.88) | 14.78 (1.94) | 14.76(1.91) | ^***^ |
| Age (W2) | 16.76(1.91) | 16.70(1.86) | 16.45 (1.94) | 16.87 (1.79) | 16.71(1.87) | 16.88(1.83) | 16.06(1.79) | 16.26 (1.78) | 16.51 (1.91) | 16.39(1.85) | ^*^ |
| Age (W3) | 18.30(1.86) | 18.21(1.84) | 18.03 (1.93) | 18.39 (1.77) | 18.22(1.84) | 18.43(1.87) | 17.53(1.82) | 17.82 (1.81) | 17.98 (1.97) | 17.91(1.89) | ^*^ |
| Family Socioeconomic Status (W1) | 3.06(0.51) | 3.12(0.58) | 3.13 (0.52) | 3.08 (0.59) | 3.10(0.56) | 3.07(0.66) | 3.00(0.72) | 3.05 (0.71) | 3.01 (0.68) | 3.03(0.70) | n.s. |
| Family Socioeconomic Status (W2) | 2.88(0.61) | 3.03(0.71) | 2.97 (0.69) | 3.01 (0.69) | 3.00(0.68) | 2.71(0.75) | 2.95(0.76) | 2.93 (0.73) | 2.76 (0.80) | 2.85(0.76) | ^*^ |
| Family Socioeconomic Status (W3) | 2.81(0.71) | 3.09(0.69) | 3.02 (0.67) | 3.02 (0.74) | 3.01(0.70) | 2.69(0.78) | 2.84(0.80) | 2.85 (0.79) | 2.71 (0.79) | 2.79(0.79) | ^***^ |
| General health (W1) | 4.07(0.78) | 4.10(0.76) | 4.12 (0.83) | 4.07 (0.71) | 4.09(0.76) | 3.95(0.78) | 3.97(0.80) | 3.95 (0.84) | 3.96 (0.74) | 3.96(0.79) | ^*^ |
| General health (W2) | 3.86(0.86) | 3.94(0.83) | 3.96 (0.87) | 3.89 (0.81) | 3.92(0.84) | 3.80(0.93) | 3.86(0.85) | 3.93 (0.89) | 3.72 (0.85) | 3.83(0.88) | n.s. |
| General health (W3) | 3.74(0.88) | 3.76(0.80) | 3.90 (0.83) | 3.65 (0.80) | 3.75(0.82) | 3.73(0.77) | 3.68(0.91) | 3.84 (0.88) | 3.56 (0.81) | 3.70(0.86) | n.s. |
| **Predictors** |  |  |  |  |  |  |  |  |  |  |  |
| Family support (W1) | 4.15(0.63) | 4.12(0.59) | 4.08 (0.57) | 4.17 (0.62) | 4.12(0.60) | 3.82(0.61) | 3.94(0.62) | 3.89 (0.58) | 3.89 (0.66) | 3.89(0.62) | ^***^ |
| Family support (W2) | 4.03(0.71) | 3.98(0.74) | 4.12 (0.68) | 3.91 (0.76) | 4.00(0.74) | 3.87(0.74) | 3.85(0.74) | 3.93 (0.68) | 3.77 (0.79) | 3.85(0.74) | ^*^ |
| Family support (W3) | 4.00(0.72) | 4.00(0.75) | 3.99 (0.69) | 3.96 (0.78) | 3.97(0.75) | 3.85(0.76) | 3.86(0.76) | 3.93 (0.73) | 3.78 (0.79) | 3.86(0.76) | ^+^ |
| Attendance to religious place (W1) | 100(95.24) | 235(88.68%) | 140(85.89%) | 195(94.20%) | 335(90.54%) | 149(87.65%) | 200(84.39%) | 182(84.65%) | 167(86.98%) | 349(85.75%) | ^*^ |
| Attendance to religious place (W2) | 67(89.33%) | 170(85.43%) | 94(84.68%) | 141(87.58%) | 239(86.28%) | 94(75.81%) | 156(78.79%) | 125(73.96%) | 125(81.70%) | 253(77.85%) | ^**^ |
| Attendance to religious place (W3) | 63(81.82%) | 165(75.69%) | 92(74.19%) | 136(79.53%) | 230(76.92%) | 94(70.68%) | 134(67.68%) | 107(64.46%) | 121(73.33%) | 231(69.16%) | ^*^ |
| Religious Support (W1) | 3.57(1.11) | 3.63(1.13) | 3.60 (1.17) | 3.62 (1.09) | 3.61(1.12) | 3.96(0.94) | 4.16(0.87) | 4.01 (0.90) | 4.15 (0.91) | 4.07(0.91) | ^***^ |
| Religious Support (W2) | 3.58(0.98) | 3.55(1.00) | 3.69 (0.81) | 3.49 (1.08) | 3.56(1.00) | 3.74(1.02) | 3.94(0.94) | 3.98 (0.87) | 3.76 (1.06) | 3.87(0.97) | ^***^ |
| Religious Support (W3) | 3.32(1.30) | 3.50(1.12) | 3.53 (1.12) | 3.34 (1.20) | 3.42(1.17) | 3.93(0.83) | 3.87(1.00) | 3.97 (0.96) | 3.83 (0.89) | 3.89(0.92) | ^**^ |
| School Support (W1) | 4.12(0.66) | 4.16(0.59) | 4.11 (0.65) | 4.18 (0.57) | 4.15(0.61) | 3.91(0.69) | 4.01(0.71) | 3.97 (0.72) | 3.97 (0.69) | 3.97(0.70) | ^***^ |
| School Support (W2) | 4.15(0.74) | 4.05(0.79) | 4.25 (0.76) | 3.98 (0.77) | 4.08(0.78) | 3.78(0.90) | 3.84(0.82) | 3.86 (0.85) | 3.76 (0.85) | 3.81(0.85) | ^***^ |
| School Support (W3) | 4.00(0.87) | 3.90(0.87) | 3.89 (0.86) | 3.95 (0.88) | 3.93(0.87) | 3.80(0.89) | 3.68(0.90) | 3.70 (0.88) | 3.76 (0.91) | 3.73(0.89) | ^**^ |
| **Outcomes** |  |  |  |  |  |  |  |  |  |  |  |
| Depressive symptoms (W1) | 1.82(0.71) | 1.81(0.78) | 1.64 (0.70) | 1.94 (0.78) | 1.81(0.76) | 1.74(0.67) | 1.86(0.76) | 1.77 (0.69) | 1.86 (0.77) | 1.81(0.73) | n.s. |
| Depressive symptoms (W2) | 1.95(0.88) | 1.86(0.79) | 1.61 (0.69) | 2.08 (0.85) | 1.89(0.81) | 1.96(0.83) | 1.97(0.84) | 1.76 (0.74) | 2.20 (0.88) | 1.97(0.84) | n.s. |
| Depressive symptoms (W3) | 2.21(0.89) | 2.06(0.80) | 1.90 (0.80) | 2.24 (0.81) | 2.10(0.82) | 2.07(0.78) | 2.22(0.86) | 1.98 (0.78) | 2.34 (0.85) | 2.17(0.84) | n.s. |
| Suicide Ideation (W1) [*n* (%)] | 8(7.34%) | 30(11.15%) | 12 (7.45%) | 26(12.50%) | 38(10.05%) | 17(9.94%) | 21(8.86%) | 19(9.05%) | 19(9.95%) | 38(9.31%) | n.s. |
| Suicide Ideation (W2) [*n* (%)] | 13(16.88%) | 22(10.89%) | 8 (7.21%) | 27(16.46%) | 35(12.41%) | 14(11.02%) | 26(13.13%) | 15(8.88%) | 25(16.45%) | 41(12.50%) | n.s. |
| Suicide Ideation (W3) [*n* (%)] | 13(16.05%) | 36(16.14%) | 16(12.60%) | 33(19.19%) | 49(15.91%) | 22(16.18%) | 33(16.42%) | 17(10.18%) | 38(22.89%) | 55(16.18%) | n.s. |

*Note.* FA = Filipino Americans; KA = Korean Americans; Mean (*SD*) for continuous variables or sample number (percentage) for categorical variables. Asterisks under “All FAs vs. All KAs” category indicate significant overall ethnic group differences across study variables. Missing cases were included in calculating the sample percentage for categorical variables.

^***^ *p* < .001. ^**^ *p* < .01. ^*^ *p* < .05. ^+^ *p* < .1

**Table S2**

*Correlations Between Study Variables at Wave 1*

|  | (1) | (2) | (3) | (4) | (5) | (6) | (7) | (8) | (9) | (10) |
| --- | --- | --- | --- | --- | --- | --- | --- | --- | --- | --- |
| (1) Developmental stages | — | −0.16^**^ | 0.02 | −0.15^**^ | −0.05 | −0.08 | −0.08 | −0.17^**^ | 0.26^***^ | 0.16^**^ |
| (2) Nativity | 0.00 | — | −0.04 | −0.05 | 0.01 | 0.10 | 0.07 | 0.11^*^ | 0.08 | −0.02 |
| (3) Biological sex | 0.10 | −0.02 | — | −0.03 | 0.01 | 0.00 | 0.00 | 0.08 | 0.06 | 0.02 |
| (4) Family SES | −0.05 | 0.06 | −0.04 | — | 0.16^**^ | 0.17^***^ | 0.13^**^ | 0.14^**^ | −0.08 | −0.03 |
| (5) General health | 0.00 | 0.02 | −0.03 | 0.18^***^ | — | 0.26^***^ | 0.19^***^ | 0.28^***^ | −0.23^***^ | −0.08 |
| (6) Family support | 0.03 | −0.03 | 0.07 | 0.04 | 0.15^**^ | — | 0.34^***^ | 0.33^***^ | −0.14^**^ | −0.03 |
| (7) Religious support | −0.04 | 0.02 | 0.01 | 0.02 | 0.10 | 0.15^**^ | — | 0.33^***^ | −0.18^***^ | −0.07 |
| (8) School support | −0.04 | 0.03 | 0.06 | 0.08 | 0.18^***^ | 0.27^***^ | 0.33^***^ | — | −0.26^***^ | −0.09 |
| (9) Depressive symptoms | 0.19^***^ | −0.01 | 0.19^***^ | −0.19^***^ | −0.33^***^ | −0.19^***^ | −0.19^***^ | −0.14^**^ | — | 0.38^***^ |
| (10) Suicidal thoughts | −0.03 | 0.06 | 0.08 | −0.07 | −0.06 | −0.13^*^ | −0.09 | −0.06 | 0.40^***^ | — |

*Note.* Correlations below the diagonal are for Filipino Americans; those above the diagonal are for Korean Americans. Developmental stages (0 = early adolescence, 1 = middle adolescence); Nativity (0 = foreign-born, 1 = U.S.-born); Biological sex (0 = male; 1 = female); ^*^*p* < 0.05, ^**^*p* < 0.01, ^***^*p* < 0.001

**Table S3**

*Correlations Between Study Variables at Wave 2*

|  | (1) | (2) | (3) | (4) | (5) | (6) | (7) | (8) | (9) | (10) |
| --- | --- | --- | --- | --- | --- | --- | --- | --- | --- | --- |
| (1) Developmental stages | — | −0.17^**^ | 0.08 | −0.21^***^ | −0.10 | 0.01 | −0.06 | −0.14^*^ | 0.14^*^ | 0.08 |
| (2) Nativity | 0.06 | — | −0.06 | 0.16^**^ | 0.03 | −0.01 | 0.03 | 0.10 | 0.01 | 0.03 |
| (3) Biological sex | 0.08 | −0.02 | — | −0.11^*^ | −0.12^*^ | −0.10 | −0.06 | −0.12 | 0.26^***^ | 0.11^*^ |
| (4) Family SES | −0.04 | 0.10 | 0.02 | — | 0.14^*^ | 0.15^**^ | 0.12^*^ | 0.08 | −0.17^**^ | −0.09 |
| (5) General health | −0.12^*^ | 0.04 | −0.04 | 0.16^**^ | — | 0.28^***^ | 0.21^***^ | 0.21^***^ | −0.32^***^ | −0.09 |
| (6) Family support | 0.04 | −0.03 | −0.15^*^ | 0.10 | 0.16^*^ | — | 0.22^***^ | 0.21^***^ | −0.19^***^ | −0.09 |
| (7) Religious support | −0.11 | −0.03 | −0.10 | −0.09 | 0.15^*^ | 0.22^***^ | — | 0.41^***^ | −0.25^***^ | −0.01 |
| (8) School support | 0.08 | −0.08 | −0.17^**^ | 0.00 | 0.17^**^ | 0.25^***^ | 0.24^***^ | — | −0.36^***^ | −0.18^**^ |
| (9) Depressive symptoms | 0.16^**^ | −0.06 | 0.28^***^ | −0.14^*^ | −0.39^***^ | −0.29^***^ | −0.27^***^ | −0.24^***^ | — | 0.50^***^ |
| (10) Suicidal thoughts | −0.06 | −0.09 | 0.14^*^ | −0.22^***^ | −0.23^***^ | −0.23^***^ | −0.21^***^ | −0.14^*^ | 0.53^***^ | — |

*Note.* Correlations below the diagonal are for Filipino Americans; those above the diagonal are for Korean Americans. Developmental stages (0 = early adolescence, 1 = middle adolescence); Nativity (0 = foreign-born, 1 = U.S.-born); Biological sex (0 = male; 1 = female); ^*^*p* < 0.05, ^**^*p* < 0.01, ^***^*p* < 0.001

**Table S4**

*Correlations Between Study Variables at Wave 3*

|  | (1) | (2) | (3) | (4) | (5) | (6) | (7) | (8) | (9) | (10) |
| --- | --- | --- | --- | --- | --- | --- | --- | --- | --- | --- |
| (1) Developmental stages | — | −0.20^***^ | 0.05 | −0.13^*^ | −0.06 | −0.12^*^ | −0.01 | −0.03 | 0.05 | 0.03 |
| (2) Nativity | −0.01 | — | −0.06 | 0.09 | −0.03 | 0.01 | −0.07 | −0.03 | 0.09 | 0.01 |
| (3) Biological sex | 0.07 | −0.02 | — | −0.09 | −0.16^**^ | −0.10 | 0.03 | −0.08 | 0.21^***^ | 0.17^**^ |
| (4) Family SES | 0.00 | 0.17^**^ | 0.00 | — | 0.21^***^ | 0.16^**^ | 0.10 | 0.06 | −0.16^**^ | −0.08 |
| (5) General health | −0.08 | 0.01 | −0.15^**^ | 0.05 | — | 0.19^***^ | 0.15^**^ | 0.13^*^ | −0.29^***^ | −0.16^**^ |
| (6) Family support | 0.01 | −0.01 | −0.02 | 0.07 | 0.16^**^ | — | 0.25^***^ | 0.13^*^ | −0.20^***^ | −0.14^*^ |
| (7) Religious support | −0.12 | 0.05 | −0.08 | 0.02 | 0.17^*^ | 0.20^**^ | — | 0.43^***^ | −0.14^*^ | −0.14^*^ |
| (8) School support | −0.11 | −0.05 | 0.03 | −0.05 | 0.09 | 0.26^***^ | 0.21^**^ | — | −0.16^**^ | −0.10 |
| (9) Depressive symptoms | 0.04 | −0.08 | 0.21^***^ | −0.08 | −0.25^***^ | −0.32^***^ | −0.22^***^ | −0.28^***^ | — | 0.39^***^ |
| (10) Suicidal thoughts | 0.01 | 0.01 | 0.09 | −0.07 | −0.17^**^ | −0.26^***^ | −0.18^**^ | −0.23^***^ | 0.45^***^ | — |

*Note.* Correlations below the diagonal are for Filipino Americans; those above the diagonal are for Korean Americans. Developmental stages (0 = early adolescence, 1 = middle adolescence); Nativity (0 = foreign-born, 1 = U.S.-born); Biological sex (0 = male; 1 = female); ^*^*p* < 0.05, ^**^*p* < 0.01, ^***^*p* < 0.001

**Figure S1.**

*Two-Way Interaction Effect Between School Support (Within) and Developmental stages on Depressive Symptoms among Korean American Adolescents*


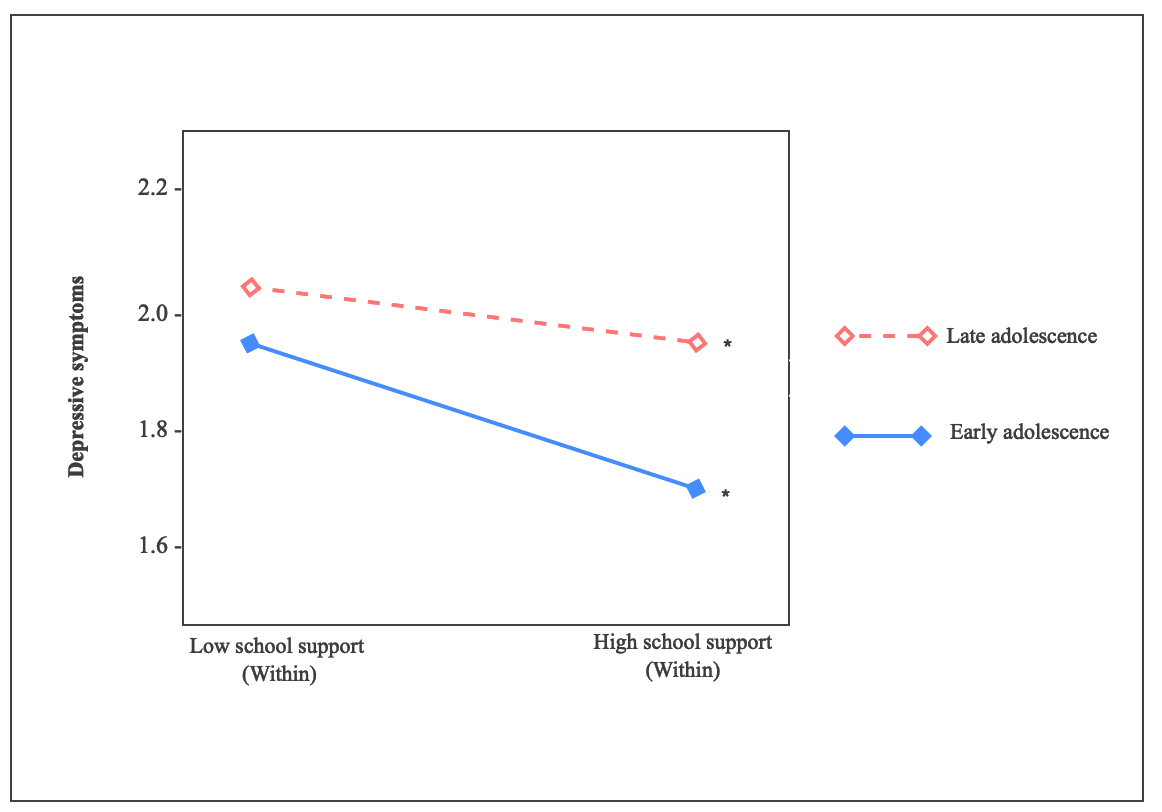


Early adolescence

Middle adolescence

*Note.* School Support (Within) = the within-subjects effect of school support. Asterisk (^*^) indicates the significance of the slope (*p* < .05).

**Figure S2.**

*Two-Way Interaction Effect Between School Support and Developmental stages on Suicidal Thoughts among Filipino American Adolescents*

**
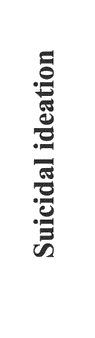
***
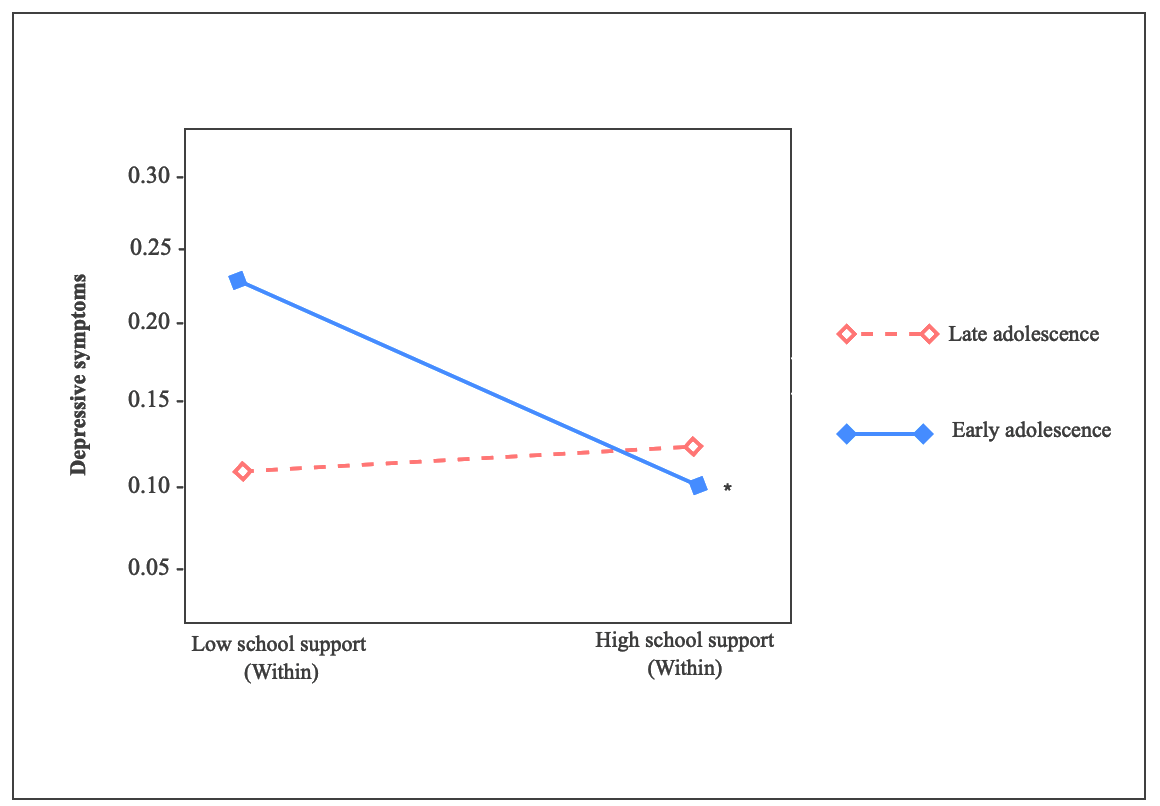
 Note.* School Support (Within) = the within-subjects effect of school support. Asterisk (^*^) indicates the significance of the slope (*p* < .05).

**Probability of Suicidal Thoughts**

Middle adolescence

Early adolescence

**Figure S3.**

*Three-Way Interaction Effect Between Religious Support, Wave, and Developmental stages on Depressive Symptoms Among FA Adolescents*

*
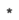

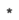

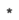
*
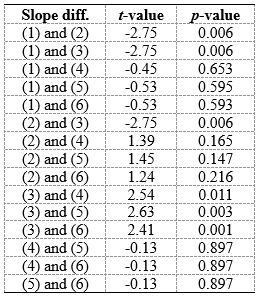
*
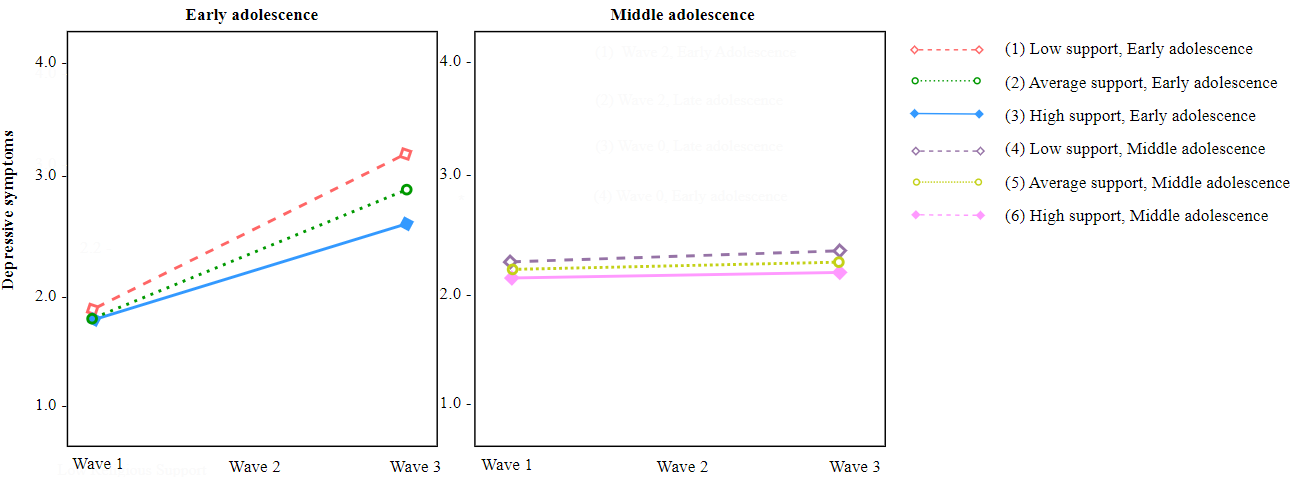
*

*Note.* Asterisk (^*^) indicates the significance of the slope (*p* < .05)

# 
